# Hemp Seed Fermented by *Aspergillus oryzae* Attenuates Lipopolysaccharide-Stimulated Inflammatory Responses in N9 Microglial Cells

**DOI:** 10.3390/foods11121689

**Published:** 2022-06-09

**Authors:** Zeyuan Wang, Lehao Wu, Dongmei Fu, Yan Zhang, Chunzhi Zhang

**Affiliations:** 1 School of Biological Engineering, Dalian Polytechnic University, Dalian 116034, China; zeyuanwang@sjtu.edu.cn (Z.W.); fudm@dlpu.edu.cn (D.F.); 2 School of Pharmacy, Shanghai Jiao Tong University, Shanghai 200240, China; wulehaogo@sjtu.edu.cn

**Keywords:** hemp seed, fermentation, anti-neuroinflammation, NF-κB, MAPKs, PI3K/Akt, ROS

## Abstract

The objective of our present work was to explore the possible enhanced anti-neuroinflammatory ability of *Aspergillus oryzae* fermented hemp seed in lipopolysaccharide (LPS)-stimulated N9 microglial cells and elucidate its underlying mechanism. The water extract of hemp seed was fermented by *Aspergillus oryzae*. LPS-stimulated N9 microglial cells were employed for the inflammatory cell model. The release of nitric oxide (NO) was determined by Griess assay. The cytokines and inflammatory mediator expression were measured by qPCR and ELISA. The phosphorylated key signaling proteins, including nuclear factor-κB (NF-κB), mitogen-activated protein kinases (MAPKs), and phosphatidylinositol 3-kinase (PI3K/Akt), were quantified by western blot analysis. The production of intracellular reactive oxygen species (ROS) was measured by DCFH oxidation. Fermented hemp seed (FHS) reduced NO production by downregulating inducible nitric oxide synthase (iNOS) expression in LPS-stimulated N9 microglial cells. FHS treatment decreased LPS-stimulated expression of inflammatory cytokines either on mRNA or protein levels. Moreover, FHS inhibited LPS-stimulated phosphorylation of NF-κB, MAPKs, and PI3K/Akt signaling pathways. Furthermore, FHS significantly reduced the ROS production in the cells. It was concluded that FHS exerted its anti-neuroinflammatory activities by suppressing ROS production, thus inhibiting NF-κB, MAPKs, and PI3K/Akt activation, consequently decreasing the expression levels of inflammatory mediators and cytokines.

## 1. Introduction

Microglia are the essential immune-responsive cells in the central nervous system (CNS). They are critically important in the immune surveillance, host defense, and tissue repair in the CNS [1]. Activated microglia release reactive oxygen species (ROS), nitric oxide (NO), and pro-inflammatory cytokines, including interleukin-1β (IL-1β), interleukin-6 (IL-6), and tumor necrosis factor α (TNF-α), in response to CNS injury [2]. These inflammatory cytokines and mediators have physiological functions that include neurogenesis, neuronal survival, and synaptic plasticity [3]. However, the excess production and release of cytokines may cause neuroinflammation-related disorders [4], such as multiple sclerosis [5], Parkinson (PD) [6], and Alzheimer’s disease (AD) [7]. Therefore, suppressing microglia activation is a crucial approach for treating neurological diseases accompanied by inflammatory processes.

In recent years, microbial fermentation has been applied to enhance the functionality of some medicinal and edible plants. For example, *Lactibacillus plantarum* and *Saccharomyces cerevisiae* fermented guava leaves demonstrate anti-inflammatory effects [8]. It has been reported that ginger fermented by *Monascus pilosus* shows enhanced anti-inflammatory activity [9]. A water extract of red ginseng improved anti-inflammatory and antinociceptive activities after fermentation with *Bifidobacterium longum* [10]. These findings imply that the proper option of beneficial microbe could improve the desired functionality of certain medicinal and edible plants.

Hemp seed (Fructus cannabis, the dried fruit of *Cannabis sativa* L.) is a typical medicinal and edible plant [11]. It contains rich vegetable protein, dietary fiber, and polyunsaturated fatty acids and is widely used in foods and animal feeds [11,12]. Hemp seed, with its laxative effects, has been recorded in Chinese Pharmacopoeia [13]. Recent studies have proved the various pharmacological effects of hemp seed on the cardiovascular system [13,14], CNS [15], and immune system [16]. In the present study, we fermented hemp seed by *Aspergillus oryzae* and compared the anti-inflammatory properties before and after fermentation using an inflammatory model system of LPS-stimulated N9 microglial cell model. We further explored the underlying molecular functions of the anti-neuroinflammatory action of the fermented hemp seed (FHP). The findings of this study will simultaneously facilitate the process of fermentation and provide more medicinal value to edible and medicinal plants.

## 2. Materials and Methods

### 2.1. Chemicals and Reagents

D-Glucose was purchased from Kermel Chemical Co. (Tianjin, China). Agar powder was purchased from Aoboxing Bio-tech Co. (Beijing, China). Hemp seed was purchased from Dalian Hongdetang Pharmacy (Dalian, China). Potatoes were purchased at a farmer’s market (Dalian, China). Lipopolysaccharide (LPS), MTT powder, and parthenolide (PAR) were purchased from Sigma-Aldrich (St. Louis, MO, USA). The NO and ROS detection kits were purchased from Beyotime Biotechnology (Jiangsu, China). The IL-1β ELISA kit and all cultural media were purchased from Thermo Fisher Scientific (Grand Island, NY, USA). The TNF-α ELISA kit was obtained from Multi Sciences (Lianke) Biotechnology (Hangzhou, China). The IL-6 ELISA kit was purchased from Neobioscience Technology Co., Ltd. (Shenzhen, China). Nitrocellulose membrane was purchased from Whatman Protran (Dassel, Germany). Antibodies for iNOS (13120S), p-JNK (4668S), JNK (9252S), p-ERK (9101S), ERK (4695S), p-P38 (4511S), P38 (9212S), p-P65 (3033S), P65 (8242S), p-Akt (4060S), Akt (9272S), and β-actin (8457S) were purchased from Cell Signaling Technology (Beverley, MA, USA). IRDye^®^ 800CW secondary antibodies were purchased from LICOR (Lincoln, NE, USA). 

### 2.2. Extract Preparation and Fermentation

**Scheme 1:**
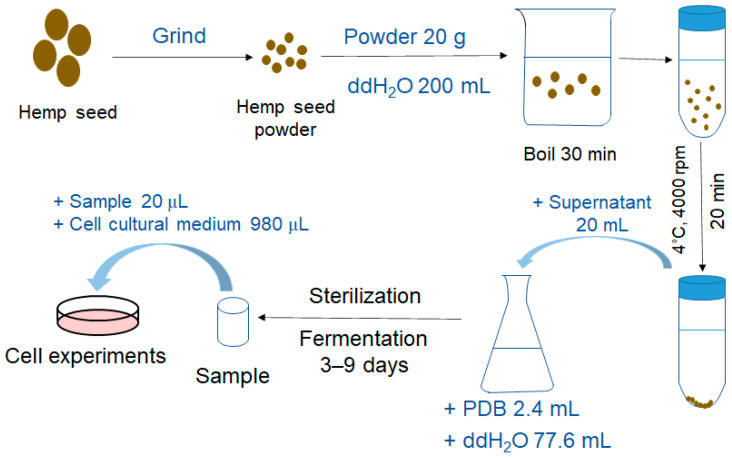
Workflow of preparation and fermentation of “hemp seed juice”.

After grinding the hemp seed, the powder was boiled in water for 30 min (at the ratio of 1:10, *w*/*v*) to make “hemp seed juice”, which was used for the experiments immediately. *Aspergillus oryzae*, obtained from the Bioengineering Institute of Dalian Polytechnic University (Dalian, China), was inoculated on the potato dextrose agar slants and cultivated at 28 °C for 7 days. The species was added to the 2.4% (*v*/*v*) fresh potato dextrose broth (PDB) involving 20% (*v*/*v*) hemp seed juice for subsequently submerged incubation at 28 °C for 9 days, referring to the fermentation time in the literature [17,18,19]. The supernatants were collected from day 3 to day 9 and referred to as “FHS”. Unfermented hemp seed involving medium, unfermented only PDB medium, and fermented regular PDB medium’s supernatants were gathered under the same conditions and named as “HS”, “PDM”, and “FP”, respectively. The workflow of preparation and fermentation of “hemp seed juice” was shown in Scheme 1.

### 2.3. Cell Culture

The N9 microglial cells were purchased from China Cell Line Bank (Beijing, China). Cells were cultured in Iscove’s Modified Dlubecco’s Medium and supplied with 10% fetal bovine serum (FBS), penicillin (100 U/mL), and streptomycin (100 mg/mL). Cells grown in a 37 °C incubator were supplied with 5% CO_2_.

### 2.4. MTT Assay

Cells were plated onto 96-well plates (1 × 10^4^ cells/well) and grown overnight. FHS, FP, HS, or PDM extracts (at the ratio of 1:50, *v*/*v*) were added into the well, respectively. MTT reagent was dissolved in double-distilled water. After 24 h, MTT solution (10 μL, 5 mg/mL) was added into the wells, and then cells were cultivated for another 4 h. After discarding the supernatant, each well was added by DMSO (100 μL). Finally, the optical density (OD) was detected at 490 nm by using Flexstation 3 (Molecular Devices, Silicon Valley, CA, USA). The mean value of the BL group was served as the control, and the cell viability was calculated as follows: cell viability (%) = OD_sample_/OD_control_ × 100%.

### 2.5. Bioassay for NO Production

Cells were plated onto 96-well plates (4 × 10^4^ cells/well) and grown overnight. The cells were precultured with either FHS, FP, HS, or PDM extracts (at the ratio of 1:50, *v*/*v*) or PAR (10 μM, positive control) for 1 h, and then stimulated with LPS (100 ng/mL) for another 24 h. The supernatant was collected and mixed with Griess reagents. After 15 min, the amount of released nitrite was measured at 540 nm by using Flexstation 3. The nitrite was calculated as follows: nitrite = (OD − 0.0508) ÷ 0.0076.

### 2.6. Total RNA Extraction and Real-Time PCR

Cells were plated onto 12-well plates (5 × 10^5^ cells/well) and grown overnight. The cells were then cultured with either FHS, FP, HS, or PDM extracts collected on Day 7 (at the ratio of 1:50, *v*/*v*) or PAR for 1 h. LPS (100 ng/mL) was added for stimulating with another 4 h. Subsequently, Trizol (Invitrogen, Carlsbad, CA, USA) was applied for the extraction of mRNA in the cells. mRNA was reverse transcribed into complementary DNA (cDNA) by the reverse transcription kit (A3500, Promega, Madison, WI, USA). cDNA was then performed to Real-Time PCR, and the transcripts of iNOS, TNF-α, IL-6, and IL-1β was measured by SYBR^®^ Green Realtime PCR Master Mix (TOYOBO, Osaka, Japan). The primers and quantification of this experiment were accomplished as described previously [20].

### 2.7. ELISA Assay

Cells were plated onto 24-well plates (2 × 10^5^ cells/well) and grown overnight. Then the cells were cultured with either FHS, FP, HS, or PDM extracts collected on Day 7 (at the ratio of 1:50, *v*/*v*) or PAR for 1 h, and then stimulated with LPS (100 ng/ mL) for another 24 h. The supernatants were gathered, and then the protein expression of IL-1β, IL-6, and TNF-α was calculated by the ELISA kit.

### 2.8. Western Blot Analysis

Cells were plated onto 6-well plates (8 × 10^5^ cells/well) and grown overnight. The cells were then cultured with either FHS extracts collected on Day 7 (at the ratio of 1:50, *v*/*v*) or PAR for 1 h, followed by stimulating of LPS (100 ng/mL), with another 24 h for iNOS, 15 min for MAPKs and PI3K/Akt, and 10 min for NF-κB, respectively. Cells were lysed with SDS-PAGE Sample Loading Buffer (Yeasen, Shanghai, China) and boiled in 99 °C for 10 min. The samples were separated with the SDS–PAGE (10%) and then transferred to nitrocellulose membrane. 5% (*v*/*v*) non-fat milk was arranged to block the membranes for 60 min at room temperature. Subsequently, the primary antibodies were incubated with membranes at 4 °C, followed by corresponding secondary antibodies on the next day. Finally, the membranes were scanned on an Odyssey^®^ CLX Infrared Imaging System. The bands were quantified through the NIH Image J software (NIH, Bethesda, MD, USA).

### 2.9. Detection of Intracellular ROS Production

Cells were plated onto a 96-well plate (6 × 10^4^ cells/well) and grown overnight. Cells were treated with FHS extracts collected on Day 7 (at the ratio of 1:50, *v*/*v*) or PAR for 1 h, and then co-stimulated with LPS (100 ng/ mL) for 4 h. The culture supernatant was deserted, and DCFH-DA (100 μL) was then added into each well for 20 min. The cells were washed with phosphate-buffered solution twice. Flexstation 3 was used to measure the fluorescence at 480/530 nm.

### 2.10. Statistical Analysis

All statistical analyses were carried out by GraphPad Prism 7.0 (GraphPad, Avenida, CA, USA). Data are exhibited as means ± S.E.M (standard error of measurement). One-way ANOVA analyzed results, and *p* < 0.05 was considered significant.

## 3. Results

### 3.1. Effects of FHS on Cell Cytotoxicity and Nitrite Production in LPS-Stimulated N9 Microglial Cells

**Figure 1:**
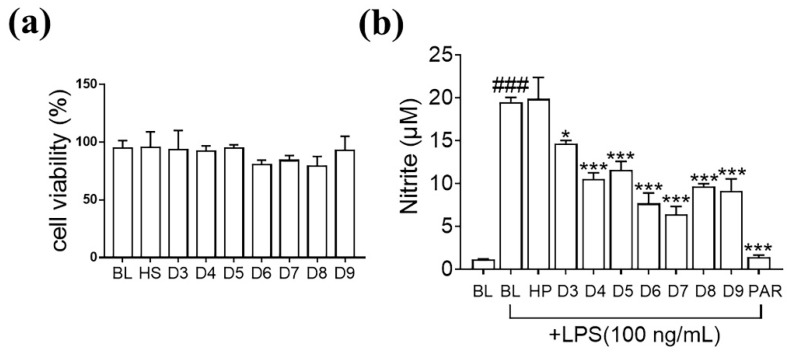
Effects of HS and FHS on cytotoxicity and nitrite production in LPS-stimulated N9 microglia. (**a**) Cell viability was examined by MTT assay. Cells were cultured with HS and different days of FHS (1:50, *v*/*v*) for 24 h (*n* = 3). (**b**) Nitrite from the supernatants was detected through Griess assay. Cells were incubated with HS and different fermentation days of FHS (1:50, *v*/*v*) for 1 h and then stimulated by LPS for 24 h (*n* = 3). BL, medium without the addition of extracts; HS, unfermented hemp seed-containing medium supernatant; D3-D9, different fermentation day’s supernatant of fermented hemp seed. ^###^ *p* < 0.001 vs the control group; * *p* < 0.05, *** *p* < 0.001 vs. the LPS-treated group.

It is necessary to validate the anti-neuroinflammatory activity of the sample without being toxic to cells. Therefore, we first assessed the potential cytotoxicity of FHS supernatants from different fermentation days and HS through MTT assay in N9 microglial cells. As Figure 1a showed, the FHS supernatants from different fermentation days and HS did not affect cell cytotoxicity.

Reducing LPS-stimulated NO production is one of the essential cases for resolving inflammations caused by immune cell activation [21]. In this study, the inhibitory effect of FHS supernatants from different fermentation days on NO production was measured through the model of LPS-stimulated N9 microglial cells. Treatment only with LPS significantly accelerated the nitrite generation, and FHS supernatants from different fermentation days (from Day 3 to Day 9) significantly suppressed this effect, whereas HS (Figure 1b), FP, and PDM did not (Figure S1).

Taken together, the FHS supernatant of Day 7 showed the best inhibition, and the suppressing effect of FHS supernatants on NO production was not due to cytotoxicity. Therefore, the FHS supernatant of Day 7 was chosen for further study in the following experiments.

### 3.2. Effects of FHS on iNOS mRNA and Protein Expression in LPS-Stimulated N9 Microglial Cells

**Figure 2:**
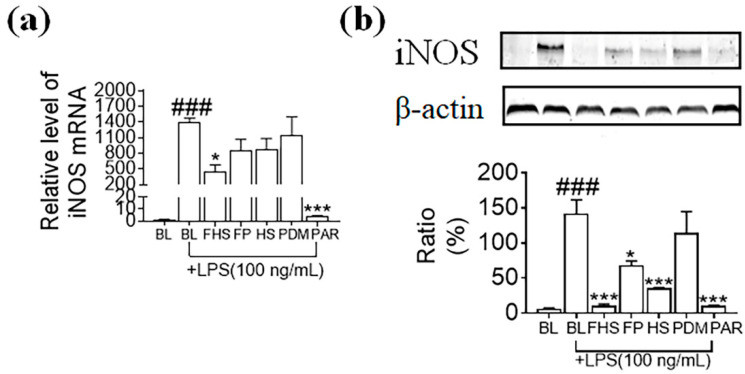
Effects of FHS, FP, HS, and PDM on iNOS at mRNA and protein expression levels. (**a**) mRNA expression of iNOS was amplified by Real-Time PCR (*n* = 3). Cells were incubated with FHS, FP, HS, and PDM for 1 h and then were incubated with LPS for another 4 h. (**b**) The protein expression of iNOS was quantified by western blot (*n* = 3). Cells were incubated with FHS, FP, HS, and PDM for 1 h, and then incubated with LPS for another 24 h. The protein expression was standardized with β-actin. BL, medium without the addition of extracts; FHS, fermented hemp seed-containing medium’s supernatant of Day 7; FP, fermented regular PDB medium’s supernatant of Day 7; HS, unfermented hemp seed-containing medium’s supernatant; PDM, unfermented regular PDB medium’s supernatant. ^###^ *p* < 0.001 vs. the control group; * *p* < 0.05, *** *p* < 0.001 vs. the LPS-treated group.

iNOS is the enzyme in charge of NO production [22]. To test the effects of FHS, FP, HS, and PDM on iNOS expression, RT-PCR and immunoblotting were employed. LPS stimulation upregulated mRNA levels of iNOS in N9 microglial cells, whereas FHS markedly attenuated this upregulation (Figure 2a). Western blot analysis demonstrated that FHS inhibited the protein expression level of iNOS, which was consistent with its effect on the mRNA level (Figure 2b). These data indicated that FHS suppressed NO production triggered with LPS by inhibiting the iNOS expression on mRNA and protein levels.

### 3.3. Effects of FHS on Pro-Inflammatory Cytokines in LPS-Stimulated N9 Microglial Cells 

**Figure 3:**
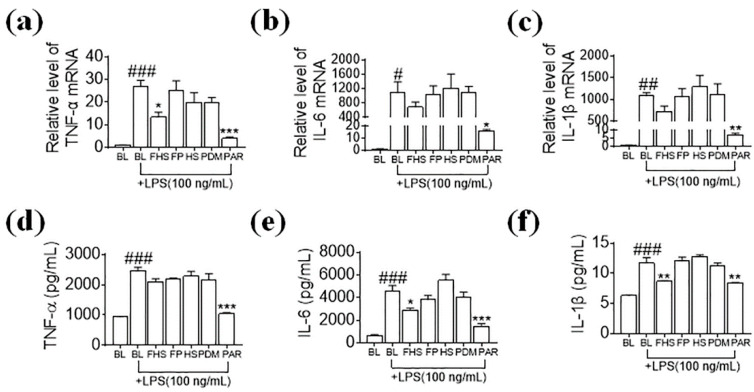
Effects of FHS on mRNA and protein expression of pro-inflammatory cytokines. (**a**–**c**) The pro-inflammatory cytokines expression on mRNA level was identified by RT-PCR analysis (*n* = 3). Cells were treated with FHS, FP, HS, and PDM for 1 h, and then cultured with LPS for another 4 h. (**d**–**f**) The protein expression of pro-inflammatory cytokines were detected by ELISA kits (*n* = 3). Cells were treated with FHS, FP, HS, and PDM for 1 h, and then cultured with LPS for another 24 h. BL, medium without the addition of extracts; FHS, fermented hemp seed-containing medium’s supernatant of Day 7; FP, fermented regular PDB medium’s supernatant of Day 7; HS, unfermented hemp seed-containing medium’s supernatant; PDM, unfermented regular PDB medium’s supernatant. ^#^ *p* < 0.05, ^##^ *p* < 0.01, ^###^ *p* < 0.001 vs. the control group; * *p* < 0.05, ** *p* < 0.01, *** *p* < 0.001 vs. the LPS-treated group.

Pro-inflammatory cytokines, such as TNF-α, IL-6, and IL-1β, are mainly produced in immune cells and typically participate in inflammation-related diseases [23]. To investigate whether FHS, FP, HS, and PDM had any effects on the production of these cytokines, we cultured N9 cells with or without the samples mentioned before in the LPS-stimulated model. Figure 3 displayed that LPS treatment significantly augmented the expression of TNF-α, IL-6, and IL-1β on the mRNA and protein levels, respectively. Pretreatment of FHS significantly attenuated the TNF-α expression on mRNA levels (Figure 3a) and slightly affected IL-6 and IL-1β expression (Figure 3b,c). The protein expression of IL-6 and IL-1β was significantly reduced by FHS treatment (Figure 3e,f), whereas TNF-α was not affected (Figure 3d). These data indicated that FHS suppressed some pro-inflammatory cytokines at the translational levels. However, FP, HS, and PDM did not exert any effects on the expression of these cytokines at either the mRNA or protein levels (Figure 3a–f).

### 3.4. Effects of FHS on NF-κB Signaling Pathway in LPS-Stimulated N9 Microglial Cells 

**Figure 4:**
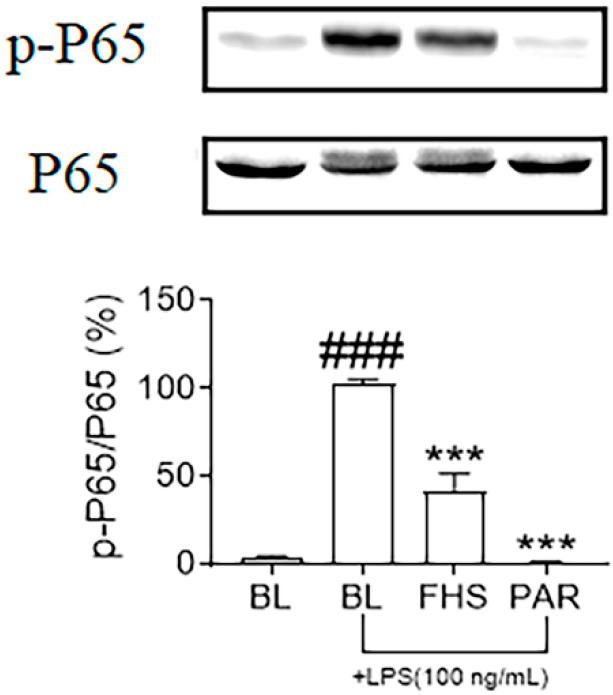
FHS suppressed the phosphorylation of P65. Cells were treated with FHS for 1 h and then incubated with LPS for another 10 min. The expression of p-P65 was analyzed by western blot and standardized with the total P65 (*n* = 3). BL, medium without the addition of extracts; FHS, fermented hemp seed-containing medium’s supernatant of Day 7. ^###^ *p* < 0.001 vs. the control group; *** *p* < 0.001 vs. the LPS-treated group.

LPS is known to activate the NF-κB signaling pathway and regulates the inflammatory mediators in microglial cells [24]. We next determined whether FHS affected the phosphorylation of the P65 subunit in the NF-κB signaling pathway by immunoblotting. Phosphorylated P65 (p-P65) increased significantly after LPS exposure and was markedly suppressed by FHS pretreatment (Figure 4). These results illustrated that FHS suppressed the activation of NF-κB in LPS-stimulated N9 microglial cells, and this mechanism might contribute to the regulation of neuroinflammatory events.

### 3.5. Effects of FHS on MAPKs and PI3K/Akt Signaling Pathways in LPS-Stimulated N9 Microglial Cells 

**Figure 5:**
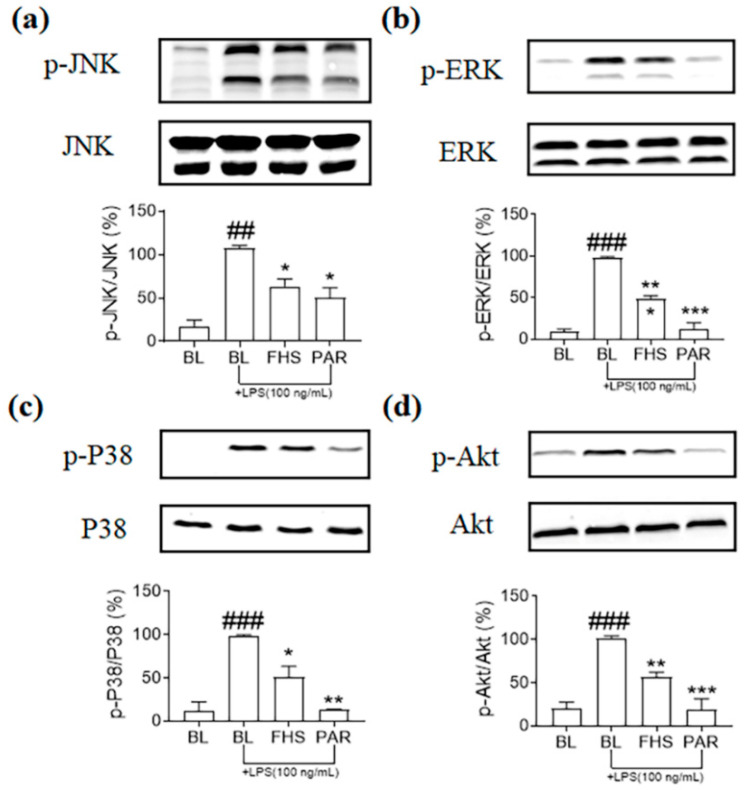
FHS suppressed the phosphorylation of key proteins in MAPKs (**a**–**c**) and PI3K/Akt (**d**) signaling pathways. Cells were incubated with FHS for 1 h and then incubated with LPS for another 15 min. p-ERK (**a**), p-JNK (**b**), p-P38 (**c**), and p-Akt (**d**) were examined by western blot and standardized with the total ERK, JNK, P38, and Akt, respectively (*n* = 3). BL, medium without the addition of extracts; FHS, fermented hemp seed-containing medium’s supernatant of Day 7. ^##^ *p* < 0.01, ^###^ *p* < 0.001 vs. the control group; * *p* < 0.05, ** *p* < 0.01, *** *p* < 0.001 vs. the LPS-treated group.

MAPKs and PI3K/AKT are another two highlighted signaling pathways involved in the LPS-stimulated neuroinflammatory response [25]. Accordingly, to evaluate those signaling pathways affected by FHS, we measured the phosphorylation of Akt and MAPKs by immunoblotting, respectively. Figure 5a–d showed that the phosphorylation of ERK 1/2 (p-ERK), JNK 1/2 (p-JNK), P38 (p-P38), and Akt (p-Akt) were augmented challenging with LPS, which were all significantly suppressed by FHS. These results illustrated that FHS exerted anti-neuroinflammatory effects through downregulating the MAPKs and PI3K/Akt signaling pathways.

### 3.6. Effects of FHS on ROS Production in LPS-Stimulated N9 Microglial Cells

**Figure 6:**
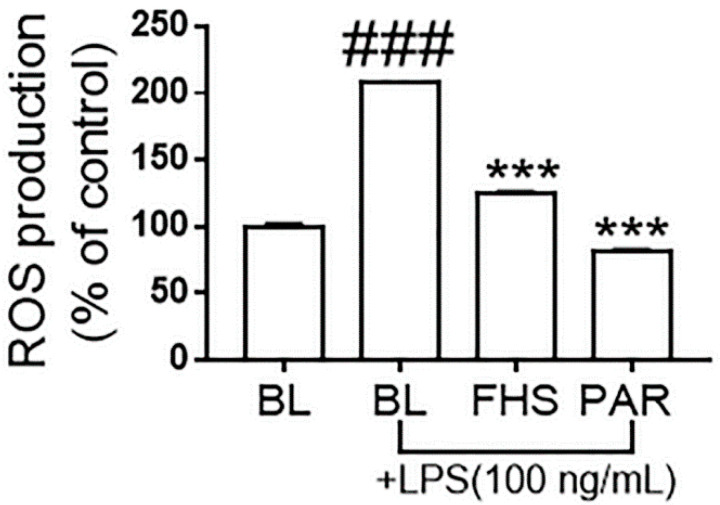
FHS suppressed ROS production. Cells were incubated with FHS for 1 h and then incubated with LPS for another 4 h. The value was calculated by dividing with control (*n* = 3). BL, medium without the addition of extracts; FHS, fermented hemp seed-containing medium’s supernatant of Day 7. ^###^ *p* < 0.001 vs. the control group; *** p < 0.001 vs. the LPS-treated group.

Our previous study has indicated that LPS-triggered ROS production in cells is closely influenced by NF-κB, MAPKs, and Akt signaling pathways, which can generate cellular damage [26]. Therefore, we assessed the antioxidant activity of FHS in LPS-stimulated N9 microglial cells by ROS measurement. ROS was determined by the ROS-sensitive fluorophore DCFH. After cells were exposed to LPS, the level of ROS increased significantly, and FHS treatment led to a reduction in fluorescence (Figure 6). These data illustrated that FHS exhibited its activity in suppressing ROS production.

## 4. Discussion

Neuroinflammation in the CNS may act as a major contributor to neuronal damage during neurological and neurodegenerative diseases [27]. Neuroinflammation is accompanied by activating microglia cells and releasing pro-inflammatory cytokines [28]. The agents that can modulate microglia activation have been advised as a feasible strategy for early-phase therapy of neurodegenerative disorders [29]. LPS-stimulated microglia are a well-established microglia-mediated neuroinflammation model. Therefore, the inhibitory effect on the expression of NO and pro-inflammatory cytokines in LPS-stimulated microglial cells implies the anti-neuroinflammatory properties of the test agents [29].

Compared to other medicinal plants, medicinal and edible plants have higher safety due to their edible properties. They have broad development potential and have attracted much attention in recent years [30,31]. Hemp seed—raw, cooked, or pressed into oil—has been used as a folk source of food for many centuries in China. Hemp seed also displayed diverse pharmacological effects, including cholesterol-lowering, immunomodulation, gastrointestinal disease treatment, and anti-aging effects [32,33,34]. Some scientific literatures reported the anti-neuroinflammatory effect of the bioactive compounds, e.g., lignanamides and pheylpropionamides in hemp seed extracts using a LPS-induced BV2 microglia model [16,35,36]. Animal studies have shown the potential beneficial effect of dietary intake of hemp seed on neurodegenerative diseases, generally associating with neuroinflammation [15,37,38]. Natural sources-derived functional foods are of great significance in the pharmaceutical manufacturing and food industries, as the desired biological activity of natural herbs may increase during the fermentation process [39]. To date, only limited efforts have been made to improve the functional potential of hemp seed through fermentation. For example, lactic acid bacteria fermentation was used to improve the antioxidant potential of hemp seed; probiotics were used to ferment a hemp seed-derived drink to enhance its prebiotic activity [40,41]. The present work investigated the effect of fermentation treatment on the anti-neuroinflammatory activity of hemp seed. 

Both *Aspergillus oryzae* and *Aspergillus niger* are important fungus in traditional fermentation and food industries. They have been used for a long time and proved to be safe [42,43,44]. In our study, we have compared the anti-neuroinflammatory effect of hemp seed with the fermentation treatment of *Aspergillus oryzae* and *Aspergillus niger*. The treatment of *Aspergillus niger* did not show any enhanced anti-inflammatory activity compared with *Aspergillus oryzae* (data not shown). Therefore, we used *Aspergillus oryzae* for further study. 

The anti-neuroinflammatory effect of hemp seed was not observed before fermentation in our in-vitro inflammatory model system. During fermentation, the anti-neuroinflammatory activity of fermented hemp seed was enhanced gradually. The most significant anti-inflammatory effect of the fermented product was observed on Day 7, as indicated by the best NO inhibition. The inhibitory effects on iNOS and some inflammatory cytokines also suggested the enhancement of anti-neuroinflammatory properties of the fermented hemp seed. The inhibitory effects of FP, HS, and PDM on iNOS were also observed at the protein level; however, we considered that this inhibition was not sufficient to affect the synthesis of NO, the downstream product. Therefore FP, HS, and PDM did not exhibit effects on NO production. During fermentation with *Aspergillus oryzae*, the plant’s chemical composition may change, and the active components of fermented hemp seed and the pharmacological correlation between them need to be further elucidated not long in the future.

We further explored the underlying mechanisms of the anti-neuroinflammatory action of the fermented products. NF-κB, MAPKs, and PI3K/Akt signaling pathways are usually correlated with the inflammatory reactions induced by LPS in immune cells [45]. NF-κB is the core regulatory agent associated with immune and neuroinflammatory responses [46]. Activating the NF-κB signaling pathway increases the release of pro-inflammatory cytokines [20] and also mediates the expression of iNOS, thereby affecting the production of NO in immune cells [47]. MAPKs and PI3K/Akt signaling pathways regulate various cellular events, including neuroinflammatory responses [48]. Previous studies have accounted that PI3K/Akt and MAPKs signaling pathways are a crucial part in pro-inflammatory cytokines expression in LPS-stimulated microglial by modulating NF-κB and other transcription factors [49,50]. Therefore, inhibition of these kinases has been considered a possible strategy for the therapy of neuroinflammatory diseases. In the present study, we found that fermented hemp seed suppressed the phosphorylation of P65, ERK 1/2, JNK 1/2, P38, and Akt in LPS-stimulated N9 microglial cells, suggesting that fermented hemp seed exerted its anti-inflammatory activities through suppressing the NF-κB, MAPKs, and PI3K/Akt signaling pathways.

**Figure 7:**
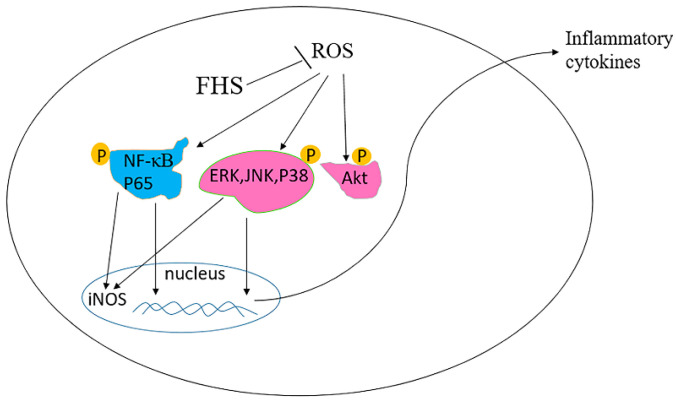
Proposed signaling pathways for FHS in LPS-stimulated N9 microglial cells.

ROS is the product of cellular metabolism. Under physiological conditions, intracellular ROS levels are finely regulated and act as messengers during cell cycle, gene expression, and cellular redox homeostasis [51]. However, excessive production of ROS can trigger oxidative stress, which leads to various disease states [52,53]. Previous studies revealed that LPS activated NF-κB, MAPKs, and PI3K/Akt signaling pathways by inducing ROS generation [26,54]. ROS released by activated microglia can increase neuronal death [55]. Thus, reducing excessive ROS production would be a promising strategy for treating neuroinflammatory disorders. In our study, fermented hemp seed significantly suppressed LPS-stimulated ROS production in N9 microglial cells. Therefore, we propose that by reducing LPS-stimulated ROS, fermented hemp seed may suppress the activation of NF-κB, MAPKs, and PI3K/Akt, which furthermore decreases the expression of inflammatory mediators and cytokines, including TNF-α, IL-1β, IL-6, and NO. The possible signaling pathway is summarized in Figure 7. 

## 5. Conclusions

The present study illustrated the significant enhancement of anti-inflammatory properties of the hemp seed after fermentation by *Aspergillus oryzae* in LPS-stimulated N9 microglial cells. Fermented hemp seed exerted its action by reducing ROS production, thus suppressing NF-κB, MAPKs, and PI3K/Akt activation and consequently inhibiting the expression levels of inflammatory mediators and cytokines. These findings support the future application of *Aspergillus oryzae* fermented hemp seed as a new potential functional food for neuroinflammatory diseases control, and it exhibits new vitality for fermentation. Further study on the isolation and characterization of anti-neuroinflammatory compounds from fermented hemp seed may guide the development of functional foods or the exploration of drug candidates for the treatment of neuroinflammatory diseases.

## Data Availability

The data used in this study are available in this article.

## Supplementary Materials

The following supporting information can be downloaded at: https://www.mdpi.com/article/10.3390/foods11121689/s1, Figure S1: Effects of FP and PDM on cytotoxicity and nitrite production in LPS-stimulated N9 microglia. Figure S2: HPLC chromatogram of HS and FHS.
