# Supplementary material for: Hemp Seed Fermented by Aspergillus oryzae Attenuates Lipopolysaccharide-Stimulated Inflammatory Responses in N9 Microglial Cells
Source: Foods. 2022 Jun 9;11(12):1689. doi: 10.3390/foods11121689 (PMC9222285; doi:10.3390/foods11121689)
Supplement: Supplementary file 1 [file foods-11-01689-s001.zip › foods-1735417-supplementary.pdf]

foods

Article

Hemp seed Fermented by *Aspergillus oryzae* Attenuates Lipopolysaccharide- stimulated Inflammatory Responses in N9 Microglial cells

Zeyuan Wang [1,†], Lehao Wu [2,†], Dongmei Fu [1], Yan Zhang [2,*], and Chunzhi Zhang [1,*]

[1] School of Biological Engineering, Dalian Polytechnic University, Dalian 116034, China; zeyuanwang@sjtu.edu.cn (Z.W.); fudm@dlpu.edu.cn (D.F.)
[2] School of Pharmacy, Shanghai Jiao Tong University, Shanghai 200240, China; wulehaogo@sjtu.edu.cn (L.W.)
* Correspondence: zhangyan_sjtu@sjtu.edu.cn (Y.Z.); zhangcz@dlpu.edu.cn (C.Z.)
† Equal contributors.

Citation: Wang, Z.; Wu, L.; Fu, D.; Zhang, Y.; Zhang, C. Hemp seed Fermented by *Aspergillus oryzae* Attenuates Lipopolysaccharide-Stimulated Inflammatory Responses in N9 Microglial Cells. *Foods* **2022**, *11*, 1689. https://doi.org/10.3390/foods11121689

Academic Editor: Alberto Cepeda Sáez

Received: 5 May 2022
Accepted: 6 June 2022
Published: 9 June 2022

Publisher's Note: MDPI stays neutral with regard to jurisdictional claims in published maps and institutional affiliations.

Copyright: © 2022 by the authors. Licensee MDPI, Basel, Switzerland. This article is an open access article distributed under the terms and conditions of the Creative Commons Attribution (CC BY) license (https://creativecommons.org/licenses/by/4.0/).

List of supporting information

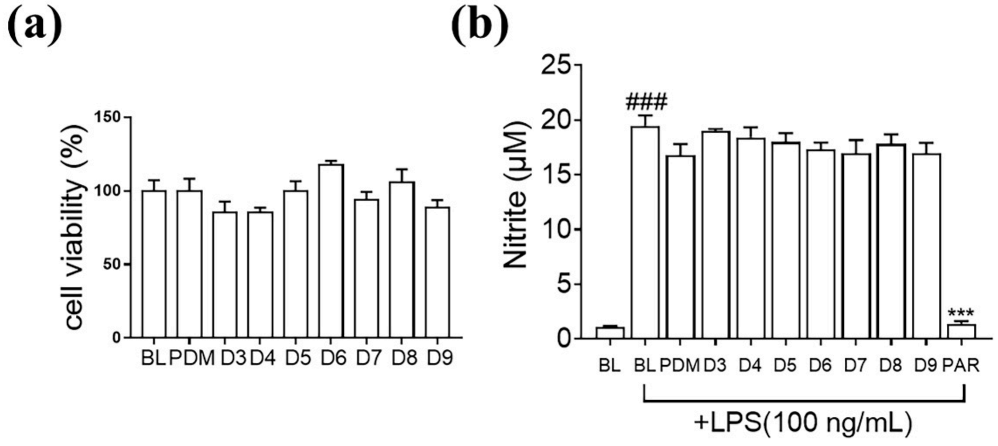

Figure S1. Effects of FP and PDM on cytotoxicity and nitrite production in LPS-stimulated N9 microglia. (**a**) Cell viability was determined by using MTT assay. Cells were incubated with PDM and different days of FP (1:50, v/v) for 24 h. (**b**) Nitrite in the supernatants was detected through Griess assay. Cells were incubated with PDM and different days of FP (1:50, v/v) for 1 h, and then incubated with LPS (100 ng/mL) for another 24 h. PAR (10 μM) was employed as a positive control. BL, medium without the addition of extracts; PDM, unfermented regular PDB medium supernatant; D3-D9, different fermentation day's supernatant of fermented regular PDB medium. The data were counted from three independent experiments and shown as the means ± S.E.M. $^{###}$ $p < 0.001$ vs the control group; * $p < 0.05$, *** $p < 0.001$ vs the LPS-treated group.

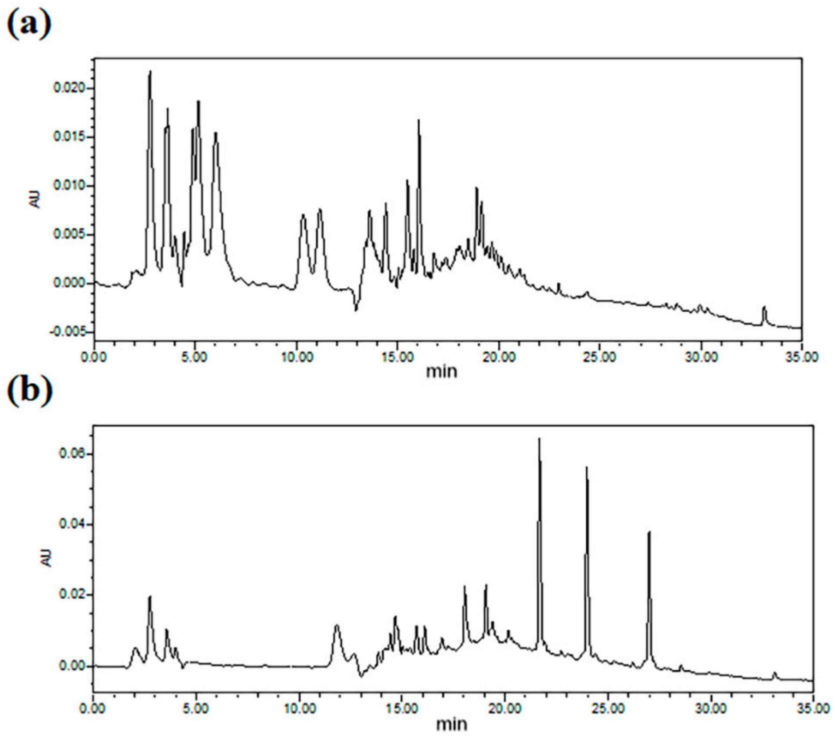

Figure S2. HPLC chromatogram of HS (**a**) and FHS (**b**). HS, unfermented hemp seed-containing medium supernatant; FHS, fermented hemp seed-containing medium's supernatant of Day 7. The analytical column was an Alliance2-C_{18} HC (250 mm × 2.1 mm i.d., 5 μm particle size). The flow rate was 0.2 mL/min, and the injection volume was 8 μL. The mobile phase was a linear gradient prepared from acetonitrile, 0.1% formic acid and ultrapure water.
